# Environmental tobacco smoke exposure in a multi-city cohort of children with asthma: Analyzing true exposure and the validity of caregiver survey

**DOI:** 10.1017/cts.2024.581

**Published:** 2024-11-11

**Authors:** Katherine McKeon, Derek Werthmann, Rebecca Straubing, Anna Rodriguez, Connie Sosnoff, Benjamin C. Blount, Ginger L. Chew, Tiina Reponen, Gary Adamkiewicz, Joy Hsu, Felicia A. Rabito

**Affiliations:** 1 Tulane University Celia Scott Weatherhead School of Public Health and Tropical Medicine, New Orleans, LA, USA; 2 Division of Laboratory Sciences, National Center for Environmental Health, Centers for Disease Control and Prevention, Atlanta, GA, USA; 3 Division of Environmental Health Science and Practice, National Center for Environmental Health, Centers for Disease Control and Prevention, Atlanta, GA, USA; 4 University of Cincinnati, Cincinnati, OH, USA; 5 T.H. Chan School of Public Health, Harvard University, Boston, MA, USA; 6 Asthma and Air Quality Branch, National Center for Environmental Health, Centers for Disease Control and Prevention, Atlanta, GA, USA

**Keywords:** Asthma, environmental tobacco smoke, information bias, healthy home, screening

## Abstract

**Introduction:**

The avoidance of asthma triggers, like tobacco smoke, facilitates asthma management. Reliance upon caregiver report of their child’s environmental tobacco smoke (ETS) exposure may result in information bias and impaired asthma management. This analysis aimed to characterize the chronicity of ETS exposure, assess the validity of caregiver report of ETS exposure, and investigate the relationship between ETS exposure and asthma attack.

**Methods:**

A secondary data analysis was performed on data from a longitudinal study of 162 children aged 7–12 years with asthma living in federally subsidized housing in three US cities (Boston, Cincinnati, and New Orleans). Data were collected at three time points over 1 year.

**Results:**

Over 90% of children were exposed to ETS (≥0.25 ng/ml of urine cotinine (UC)). Exposure was consistent over 1 year. Questionnaire data had a sensitivity of 28–34% using UC ≥0.25 ng/ml as the gold standard. High ETS exposure (UC ≥ 30 ng/ml) was significantly associated with asthma attack (aOR 2.97, 0.93–9.52, *p* = 0.07). Lower levels (UC 0.25–30 ng/ml) were not statistically significant (aOR 1.76, 0.71– 4.38, *p* = 0.22). No association was found using caregiver-reported ETS exposure.

**Conclusion:**

Relying on questionnaire data to assess children’s exposure to tobacco smoke may lead to substantial information bias. For children with asthma, incorrect characterization may substantially impact asthma morbidity.

## Introduction

The impact of environmental pollutants on health is a growing concern for public health. This paper examines a significant contaminant, environmental tobacco smoke (ETS), and addresses the challenges of assessing children’s exposure using the traditional method of survey questionnaire. Accurate exposure assessment is a considerable challenge. For many environmental hazards, exposure assessment is difficult to determine; therefore, biomarker assessments of exposure, rather than self-report, is often preferred to increase validity.

Exposure to ETS leads to a variety of adverse health consequences [1]; public officials have recognized this risk by implementing smoke-free policies in public spaces [2]. However, the extent to which these policies limit children’s exposure remains unclear. Children with asthma are particularly susceptible to the adverse effects of ETS due to their sensitivity to pulmonary insult: ETS is a known trigger of asthma attacks [3–5]. Avoiding asthma triggers is an integral component of asthma management [6]. Environmental asthma triggers like irritants (air pollutants) and allergens (dust mite, cockroach, and mold) often co-occur in the home [7,8]. Reducing a child’s exposure to these triggers can reduce asthma morbidity, improve asthma control, prevent lung function decline, and can reduce the need for medication [7].

Therefore, accurately measuring ETS exposure is an important part of asthma management. The biomarker cotinine is an objective method to estimate ETS [9]. Cotinine is the major proximal nicotine metabolite, has a half-life of approximately 15 hours [10], and is a commonly used biomarker of ETS.

Despite the established link between tobacco smoke and adverse health effects in children, an estimated 40% of US children aged 3–11 years are still exposed [1], with ETS being the primary source. ETS originates from secondhand smoke (SHS) and thirdhand smoke (THS). Smoke particles from combusted tobacco products can remain suspended in the air for extended periods of time. When a nonsmoker inhales these particles, it is considered SHS exposure [11]. Tobacco smoke particles can settle on surfaces and embed in materials such as carpet and drapery. These particles can be ingested, absorbed dermally, or inhaled when resuspended [11]. Exposure via these pathways is considered THS [11]. THS is difficult to remove [12], resulting in reservoirs of smoking contaminants. Research has found that individuals living in poverty are less able to replace smoke-embedded items and influence smoking behaviors in multiunit buildings and that children from low-income households have higher levels of THS exposure compared to children from higher-income households [13].

In practice, children’s ETS exposure is often assessed via caregiver questionnaire. Studies assessing the reliability of caregiver-reported ETS exposure show mixed results [14–18]. However, reliability (precision) should be distinguished from validity (accuracy), and correlation is not the appropriate measure to assess validity in the presence of information bias. In these situations, sensitivity is a better measure [19] of the validity of survey questionnaires. To our knowledge, only one study in the past 10 years has assessed the validity and potential for measurement error when using caregiver reports to estimate children’s ETS exposure. In that study, salivary cotinine was used as the gold standard and the authors found nearly 40% of exposed children were misclassified as non-exposed [20]. In addition to the potential for misclassification bias, another limitation to current estimates of children’s ETS exposure is that most studies measure ETS only once, despite studies reporting that one-time measurements may not accurately reflect longer-term exposure [21,22].

This analysis aimed to characterize the magnitude and chronicity of ETS exposure in a cohort of children with asthma, to assess the validity of caregiver-reported ETS exposure, and to investigate the relationship between ETS and asthma attacks using two exposure assessment methods: caregiver questionnaire and urinary cotinine (UC).

## Materials and methods

### Study design

This secondary analysis utilizes data from the Green Housing Study, a longitudinal, repeated measures cohort study conducted from 2011 to 2016 that assessed environmental risk factors on asthma morbidity [23,24]. Eligibility criteria included age 7–12 years, healthcare provider-diagnosed asthma, experiencing asthma-related symptoms (wheezing, or night-time awakenings) during the previous 6 months, caregivers reporting that the child slept in the home seven nights per week, and living in US Department of Housing and Urban Development (HUD) subsidized housing. Children were enrolled from three study sites: New Orleans, Louisiana; Boston, Massachusetts; and Cincinnati, Ohio. A convenience sample of caregivers of children with asthma was recruited via community events. The cohort included 162 children followed for 1 year. Survey and biologic data were collected in the participant’s home at three time points, baseline, six and 12 months, by trained research staff. Each home visit consisted of two data collection points (day 1 and 5 days later).

### Data collection

ETS exposure was assessed in two ways at each home visit. First, caregivers were asked: (1) “Do visitors smoke in your home?” and (2) “Do you smoke or does a household member smoke cigarillos, cigarettes, cigars, pipes, and other tobacco products?.” A positive response to either question was considered exposed. Second, a convenience spot and first morning void (FMV) urine sample were collected on either day 1 or day 5, depending on the readiness of the child. The spot sample was collected when field technicians were in the home, assuring the sample came from that child. To obtain the FMV sample, caregivers were given instructions and a sterile urine cup. Samples were stored in the home freezer until picked up by study personnel and transported to the study site’s laboratory on ice packs and stored at −80°C until shipped to the National Center for Environmental Health Division of Laboratory Sciences for analysis. The sample (either spot or FMV) with the highest cotinine level at each collection point was used in the analysis, resulting in a maximum of three samples per child. Exposure to ETS was treated as continuous and categorical. To aid comparability, we used exposure categories established by Benowitz et al. and which have been used in previous studies [16,18,25,26]. Various exposure groups were compared. An ordinal categorical variable was created: light SHS or THS (UC ≥0.05 to <0.25 ng/ml), SHS (UC ≥ 0.25 to <UC 30 ng/ml), and high SHS (UC ≥ 30 ng/ml) [25]. For the middle and high categories, due to the children’s young age, we assumed they were not active smokers, and the category labels reflect that assumption. Three binary categorical variables were also created: UC ≥ 0.05 ng/ml, UC ≥ 0.25 ng/ml, and UC ≥ 30 ng/ml. The prevalence of asthma attack in the previous 3 months was obtained via survey questionnaire and treated as a binary outcome (yes/no). Baseline covariates include child’s sex, age, household income, home type, body mass index (BMI) percentile, caregiver-reported child’s race/ethnicity, caregiver education level and marital status, and whether the child had a designated healthcare provider. For children with UC ≥30 ng/ml, a level consistent with being an active smoker, child’s age was checked to examine the non-smoker assumption [27].

### Statistical analysis

To characterize the magnitude and to examine chronicity of ETS exposure, the prevalence of ETS exposure overall and by race/ethnicity, income, and at each study visit was calculated. To quantify the consistency of UC level, the intraclass correlation coefficient (ICC) was calculated for continuous and Cochran’s Q for categorical UC. To assess the validity of questionnaire-derived ETS exposure, the sensitivity, specificity, positive predictive value (PPV), and negative predicted value (NPV) were calculated using UC cut points of ≥0.25 ng/ml and ≥30 ng/ml as the gold standards [25,28].

Semi-parametric generalized estimating equations models accounted for the repeated measures design and the skewed distribution of UC and were clustered by study site. We ran separate models for each ETS exposure method (questionnaire, UC binary, and UC ordinal variables) using a binomial distribution. Age was modeled continuously, while the other variables were modeled categorically. Household income was dichotomized at $10,000 per year. Caregiver-reported child race/ethnicity non-mutually exclusive categories were Hispanic, Black, Asian, White, and Other (defined as anything other than the previously listed categories), but were collapsed into Black vs other (defined as Other plus Hispanic, Asian, and White due to small sample size) for modeling purposes. Caregiver education was categorized as no high school degree, high school graduate, or some college, and caregiver marital status as currently married or not. BMI categories were categorized as healthy, underweight, overweight, and obese [29]. Covariates were included in adjusted models if they were associated with asthma attack (*p* ≤ 0.10).

## Results

At baseline, the median age of children was 9.53 years, 48% were female, 72% were Black, 57% had annual household incomes less than $10,000, 91% had a designated healthcare provider, and median UC was 3.06 ng/ml (IQR = 0.63–14.0 ng/ml) (Tables 1 and 2). All children had UC ≥0.05 ng/ml; 93% had UC ≥0.25 ng/ml, and 17% had UC ≥ 30 ng/ml. When treated as an ordinal variable, at baseline 7% were exposed to light SHS or THS (UC ≥0.05 – <0.25 ng/ml), 76% to SHS (UC ≥0.25– <30 ng/ml), and 17% to high SHS (UC ≥30 ng/ml). The median age of the 26 children with UC ≥30 ng/ml was 8.77 years (IQR 7.57–10.02). Pronounced differences in exposure were found by race/ethnicity and income status. At baseline, the median UC for Black children was 7.78 ng/ml (IQR 1.65–29.10) compared to 0.52 ng/ml (IQR 0.29–1.19) for Other race/ethnicity. These differences were statistically significant (Wilcoxon rank-sum *p*-value <0.001). Median UC for children from families reporting annual household income < $10,000 was 5.53 ng/ml (IQR 1.35–27.45) compared to 0.77 ng/ml (IQR 0.36–4.83) for children from households reporting income ≥$10,000. These differences were statistically significant (Wilcoxon rank-sum *p*-value of <0.001).

**Table 1.** Baseline characteristics of the study population, N = 162

(A) Baseline characteristics of study population (n = 162)			
	N (%)	n Missing	Median (IQR)
Age (years)	162	0	9.53 (8.27–11.01)
Age (years), UC ≥30 ng/ml	26(16)	0	8.77 (7.56–10.02)
Female	77(48)	0	
Child race/ethnicity*		0	
Black	117(72)		
Other	45(27)		
Designated healthcare provider for child	147(91)	1	
BMI category		0	
Healthy	89(55)		
Underweight	11(7)		
Overweight	28(17)		
Obese	34(21)		
Annual household income*		6	
< $10,000	92(57)		
≥ $10,000	64(40)		
Number of people living in home		7	4 (3–5)
Housing type		7	
Single family	69(42)		
Multi-family	86(53)		
Caregiver education		5	
No high school degree	39(24)		
High school graduate	67(41)		
Some college	51(31)		
Caregiver married	35(22)	2	
Asthma outcomes (attack in past 3 months)	48(30)	2	
**(B) ETS exposure**			
Urine cotinine (UC)		11	
UC ≥0.05– <0.25 ng/ml	11(7)		
UC ≥0.25– <30 ng/ml	114(76)		
UC ≥30 ng/ml	26(17)		
Black, UC (ng/ml)	107	10	7.78(1.65–29.10)
Other, UC (ng/ml)	44	1	0.52(0.29 – 1.19)
Asian, UC (ng/ml)	27		0.41(0.27, 0.73)
Hispanic, UC (ng/ml)	17	1	0.80(0.44, 1.29)
Non-Hispanic Black, UC (ng/ml)	105	10	8.75(1.79, 29.1)
Income < $10,000, UC (ng/ml)	84(58)	8	5.53(1.35–27.45)
Income ≥ $10,000, UC (ng/mL)	61(42)	3	0.77 *l* (0.36–4.83)
**ETS questions**			
Visitors smoked in home	47(30)	7	
Household members use tobacco products	52(32)	8	
Positive response to either ETS question	63(39)	7	

Significant differences at *p* < .0.0001.

IQR = Interquartile range; UC = urine cotinine; ETS = environmental tobacco smoke.

**Table 2.** Prevalence of children exposed to environmental tobacco smoke (ETS) at each follow-up

Exposure	Baseline (N = 151)	Month 6 (N = 140)	Month 12 (N = 118)	ICC or Cochran’s Q
Cotinine ng/ml, continuous (median, IQR)	3.06 (0.63, 14.00)	2.69 (5.33, 14.40)	1.06 (0.46, 3.27)	0.94
**Cotinine (Binary, yes vs no)**	**n (%)**	**n (%)**	**n (%)**	
UC ≥0.05 ng/ml	151(100)	140(100)	118(100)	NA
UC ≥0.25ng/ml	140(93)	132(94)	110(93)	0.38, *p* = 0.83
UC ≥30 ng/ml	26(17)	17(12)	7(6)	4.31, *p* = 0.12
**Cotinine (ordinal)**				
UC ≥0.05– <0.25 ng/ml UC ≥0.25– <30 ng/ml UC ≥30 ng/ml	11(7) 114(76) 26(17)	8(6) 115(82) 17 (12)	8(7) 103(87) 7(6)	NA
**Questionnaire variables**	***N* = 155**	***N* = 138**	***N* = 139**	
Visitors smoke in home	47(30)	37(27)	40(29)	0.14, *p* = 0.93
Household members use tobacco products	52(34)	39(33)	47(38)	1.11, *p* = 0.58
Positive response to either ETS question	63(41)	46(33)	54(39)	1.70, *p* = 0.43

ICC = intraclass correlation coefficient; UC = urine cotinine; NA = not applicable; IQR = interquartile range.

The prevalence of children exposed to ETS did not change significantly over the follow-up year for either exposure assessment method (Table 2). For continuous UC, the ICC was 0.94, indicating high continuity of ETS exposure for individual children. Treated categorically, Cochran’s Q *p*-values were 0.83 for UC ≥ 0.25 ng/ml and 0.12 for UC ≥ 30 ng/ml. For the questionnaire data, Cochran’s Q *p*-values were 0.93 for visitors smoke inside the home, 0.58 for household members use tobacco products, and 0.43 for a positive response to either question.

The sensitivity (the proportion of children identified as exposed using UC who also were identified as exposed using survey questions) was low for both questions (Table 3). Among children with UC ≥0.25 ng/ml, 28% of caregivers indicated that a visitor smoked in home. In contrast, the specificity (96%) and PPV (99%) were high and the NPV (9%) low for the visitor smoking question, suggesting that regardless of whether the caregiver responded positively or negatively about children’s ETS exposure, children were exposed to ETS at UC ≥ 0.25 ng/ml. Similar results were found for the household member tobacco use question. The sensitivity was 34%, indicating that among children who had UC ≥ 0.25 ng/ml, only 34% of caregivers reported that visitors smoked inside the home. For this question, the specificity was 96%, the PPV 99%, and the NPV 8.9%. The low sensitivities demonstrate that UC does not align with survey data responses. We then assessed validity using the highest binary UC category (UC ≥ 30 ng/ml) as the gold standard. This resulted in greater sensitivity; visitor smoke scored 71%, household member tobacco use scored 73%, and a positive response to either question scored 83%.

**Table 3.** Assessment of the validity of caregiver report of child’s exposure to environmental tobacco smoke (ETS)

Cotinine≥0.25 ng/ml	Sensitivity % (95% CI)	Specificity % (95% CI)	Positive predictive value % (95% CI)	Negative predictive value % (95% CI)
Visitors smoke in home	27.64(23.08, 32.21)	96.15(88.76,100)	99.03(97.14,100)	8.56(5.35,11.77)
Household members use tobacco products	33.92(28.88, 38.96)	95.65(87.32, 100)	99.03% (97.14, 100)	8.94(5.38, 12.51)
Yes to either question	36.59(31.67, 41.5)	92.31%(82.07, 100)	98.54% (96.53, 100)	9.30(5.76, 12.85)
**Cotinine≥30 ng/ml**				
Visitors smoke in home	70.83(56.82, 81.76)	80.12(75.6, 83.98)	33.01% (24.68, 42.56)	95.21(92.11, 97.12)
Household members use tobacco products	72.92(59, 83.43)	74.20(69.09, 78.73)	30.17 (22.57, 39.05)	94.72(91.17, 96.89)
Yes to either smoking question	83.33(70.42, 91.30)	72.05(67.1, 76.51)	29.20 (22.23, 37.29)	96.90(94, 98.42)

CI = confidence interval.

The association between ETS and asthma attacks varied by exposure assessment method (Table 4). Measured as a continuous variable, UC was not associated with asthma attack in either unadjusted or adjusted models (OR 1.01, 95% CI = 0.99–1.03; aOR 1.00, 95% CI = 0.98–1.03). Treated as an ordinal categorical variable, UC ≥ 0.25–<30 ng/ml was not statistically significant (OR 1.75, 95% CI = 0.72–4.24, *p* = 0.22; aOR 1.76, 95% CI = 0.71–4.38, *p* = 0.22). High UC, ≥30 ng/ml was significantly associated with a three-fold increase in asthma attacks (OR 3.33, 95% CI = 1.11–9.55, *p* = 0.03); however, when adjusted for age, the association attenuated (OR 2.97, 95% CI = 0.93- 9.52, *p* = 0.07). When ETS exposure was assessed using either survey question, exposure was not associated with asthma attacks in either adjusted or unadjusted models (Table 4).

**Table 4.** Association between exposure to environmental tobacco smoke (ETS) and asthma attack in the previous 3 months, *N* = 151

	Unadjusted models			Age-adjusted models		
	Odds ratio and 95% CI	*P*-values	*P*_for trend_	Odds ratio and 95% CI	*P*-values	*P*_for trend_
**Questionnaires**						
Visitors smoke in home	1.03(0.66, 1.64)	0.90		1.02(0.64-1.64)	0.95	
Household members use tobacco products	0.87(0.54, 1.39)	0.56		0.81(0.45-1.32)	0.34	
Yes to either question	0.99(0.64, 1.54)	0.97		0.92(0.53, 1.46)	0.73	
**UC, ng/ml**						
Continuous*	1.01(0.99, 1.03)	0.45		1.00(0.98, 1.03)	0.76	
UC ≥0.25 ng/ml	1.83(0.78, 4.31)	0.10		1.83 (0.75, 4.55)	0.12	
**Categorical levels of ETS**		0.09	0.03**		0.16	0.08
UC ≥0.05- <0.25 ng/ml	Reference			Reference		
UC ≥0.25 and<30 ng/ml	1.75(0.72, 4.24)	0.22		1.76(0.71, 4.38)	0.22	
UC ≥30 ng/ml	3.33 (1.12, 9.95)	0.03**		2.97(0.93, 9.52)	0.07	

ORs modeled as change in 5 ng/ml.

Significant at alpha<0.05.

UC = urine cotinine; CI = confidence interval.

## Discussion

ETS exposure was ubiquitous in this multi-city cohort. Using a cut point of UC ≥ 0.05 ng/ml, all children were exposed. Our results are consistent with findings of a study by Benowitz where 87% of hospitalized adolescents had UC ≥0.05 ng/ml [25]. The prevalence of UC > 0.25 ng/ml in our cohort is higher than that reported by Stallings where 59% of hospitalized children 0–5 years had UC > 0.25 ng/ml [30], and higher than the overall prevalence of ETS exposure (as measured by serum cotinine) by NHANES with 39% of children aged 3–11 years old [31]. The characteristics of children in our study differed from these studies in some ways (e.g., non-hospitalized cohort, different age range), thus adding to our understanding of the prevalence of ETS in US children. Despite pronounced disparities in ETS exposure by race/ethnicity and income status consistent with findings in the NHANES sample [31], neither variable was an independent risk factor for asthma attack in our study and, therefore, did not confound the relationship with UC. Housing type was not associated with asthma attacks in this analysis, though previous research has demonstrated contrasting findings on this issue [32]. In addition to ETS, allergens and air pollutants are also important when assessing asthma care. Future research should explore the impact of co-exposure to irritants and allergens in order to comprehensively understand the contribution of the home exposome on pediatric asthma.

We found little variation in an individual child’s exposure to ETS over 1 year. This is inconsistent with previous studies which report that cotinine levels vary over time; however, the populations in those studies are substantially different from those in the green housing study. A study in women undergoing in vitro fertilization (IVF) reported an ICC of 0.40 for follicular fluid cotinine over two 6–8-week IVF cycles. Similarly, in a study of children from birth to 7 years, the authors reported that UC was not constant; a single sample estimated only recent exposure from 2 to 3 days [22].

Exposure to ETS is an increasing public health concern due to the newfound dangers of THS [26]. Chemicals in THS may react with other air pollutants to become highly mutagenic secondary pollutants, such as tobacco-specific nitrosamines (TSNAs) [33,34]. Cloth THS has been found to contain a 10 times higher TSNA-to-nicotine ratio than aerosol samples, possibly indicating carcinogenic concentration [35]. While the source of ETS exposure in this cohort was unclear, persistent THS reservoirs are suspected because it is difficult to remove from fabric and building materials [12]. Accurately measuring a child’s exposure to ETS is important, particularly for children with asthma, a group at elevated risk of experiencing worsening symptoms from such exposure. We found that caregiver report of ETS exposure was not valid when using UC ≥ 0.25 ng/ml as the gold standard, consistent with previous studies. In a cohort of mother–infant dyads, mothers’ report of infant ETS exposure explained only 31% of the variance in their child’s UC. Using a cut point of 0.31 ng/ml, 76% of the infants were exposed to ETS, but only 12% of mothers reported the infant being exposed [16]. Similarly, in a cohort of middle-class families, the correlation between UC and questionnaire data was low (*r* = 0.04) [14].

The smoking questions in our study performed differently by exposure status. Using a UC cut point of 0.25 ng/ml, the sensitivity was 37%, indicating the potential for substantial information bias (63% of children with high UC misclassified as unexposed). However, the questions had high specificity (>90%) indicating accurate reporting of unexposed children (UC < 0.25 ng/ml). For public health and healthcare, sensitivity is an important measure of validity because identifying children exposed to ETS is necessary to direct intervention efforts [36]. While specificity is important to minimize false positives, in the case of exposure to toxicants like ETS, the priority is to accurately identify as many exposed individuals as possible, making sensitivity a more critical measure in this context.

Sensitivity was higher (>70%) for children with UC ≥ 30 ng/ml; however, research shows that the impact of ETS exposure on asthma occurs at low levels (UC 0.05–10 ng/ml) and can have lasting health impacts [37]. The disagreement between questionnaire and biomarker data may be because the questionnaires did not account for all possible exposure locations; in addition, the dangers of THS are a relatively new concept [38] and the questionnaire did not explicitly ask about THS; therefore, caregivers may lack knowledge about what constitutes exposure [38]. The location of ETS exposure cannot be determined in this study; however, children slept at home seven nights a week. Considering that children of this age typically spend the majority of their time either at home or in school (a smoke-free environment), combined with the high ICC of the UC, it is possible that the home is a contributing source of ETS exposure.

We did not adjust for urinary creatinine because of the age range of our study participants. Creatinine excretion rate is influenced markedly by lean body mass, and lean body mass can change markedly as children enter puberty. Thus, while urinary creatinine can help adjust for variable hydration, creatinine correction may not improve the precision of individual UC measurements in certain groups. [39]. Our approach aligns with contemporary ETS research [17,25,40].

Consistent with our findings, previous research reported that the association between ETS exposure and asthma outcomes in children varies depending on the exposure assessment method [41]. A cross-sectional study of 466 children found that caregiver-reported ETS exposure was not predictive of asthma exacerbation while salivary cotinine was [42]. In our longitudinal study, we found non-statistically significant relationships between UC and asthma attack and no association when using questionnaire-reported exposure. Consistent with our analysis of sensitivity and specificity, exposure misclassification from parental report obscured the relationship between a child’s exposure to ETS and asthma outcomes.

This study provides important information on the prevalence of ETS exposure and the relationship to pediatric asthma; it has several strengths including the longitudinal repeated measures design, multiple urine samples, and the multi-city locations. Limitations include missing UC data as the study progressed. Multiple imputation was not performed because UC was the main variable of interest. We assume missing at random because the prevalence of UC did not vary over time, as shown by the high ICC. Another potential limitation is that we assumed children were non-smokers; however, 26 children had UC high enough to be classified as active smokers (UC ≥ 30 ng/ml). Given their young age (median age 8.77 years, IQR: 7.56–10.02), we concluded they were most likely not habitual smokers and were included in the analysis. Also, generalizability beyond our study population might be limited (e.g., rural, different socioeconomic status). Related to this, the Green Housing Study (the parent study) was conducted in homes where the prevalence of smoking was high, which may explain the high PPV and low NPV, especially because these measures (and not sensitivity and specificity) are influenced by prevalence [43]. The questions may be useful in settings where the prevalence of smoking is lower.

Finally, a major limitation was that the parent study was not designed to evaluate ETS exposure validity, and smoke exposure was not assessed outside of the home. Other questions may offer greater sensitivity. However, this may not be true if low validity is due to lack of knowledge about what constitutes ETS rather than respondent bias. Other studies employed similar questions such as “Does either parent smoke?,” “Is the child exposed to ETS?,” “Do you currently smoke cigarettes, even occasionally?,” or simply used pediatric electronic health record designation; these are similar in scope to the questions in this analysis [18,20]. Emerson et al. showed the highest agreement between caregiver report and UC, but this was an interventional study with parents who identified as smokers [14].

## Conclusion

Our study adds to the literature on ETS by examining the prevalence and validity of exposure over time in a cohort of children with asthma living in subsidized urban housing. Our findings suggest that caregiver report of home ETS exposure is not sensitive in discerning true exposure of a child with asthma and that the relationship between ETS and asthma is likely underreported when relying on questionnaire data. Therefore, UC biomarkers may be useful where feasible. Research on the knowledge, attitudes, and perceptions of ETS, especially of THS (a relatively new concept), could enhance future public health initiatives [12,13]. People may not know that THS poses a threat to their child’s health [38] and that reservoirs of THS increase in toxicity over time [34,35]. Interventions to prevent THS exposure differ from SHS; therefore, further research distinguishing SHS from THS may be valuable for designing policy and public health interventions to prevent children’s exposure to ETS [13].

When addressing exposures that are not easily detectable, one might consider adopting a method similar to lead screening to identify children at high risk of exposure. Lead screening commonly takes into account factors such as zip code, the age of housing, and other epidemiological evidence [44]. Like elevated lead levels, pediatric asthma appears to cluster in specific geographic areas [45,46]. By examining hospitalization rates and housing code violations, it is possible to identify groups of children who are at risk for asthma-related complications [47]. Implementing targeted interventions in neighborhoods with higher asthma burden has been shown to effectively decrease hospitalization rates [48]. These types of interventions rely on geographic screening and neighborhood data to assess risk rather than self-report data. Implementing a similar approach to identifying ETS exposure could help reduce exposure in children who are at higher risk of developing asthma [25,32].

Our study contributes to the existing literature by demonstrating that cotinine levels provide a more reliable method for determining exposure compared to parental questionnaires, even over extended periods of time. Furthermore, our findings suggest that in this cohort of children with asthma, ETS exposure, measured via urine cotinine, remained relatively stable over 1 year, indicating chronic ETS exposure. Gaining a greater understanding of the influence that environmental factors have on human health is a critical direction for the field of public health. The significant impact of ETS exposure on asthma attacks in children clearly demonstrates this. Furthermore, given the high reliability of UC from the high ICC, the non-invasive nature of urine sampling, the importance of ETS exposure on a child’s asthma, and the high proportion of families reporting having a designated healthcare provider, our results suggest that public health officials, researchers and healthcare providers serving children with asthma may want to consider testing for ETS exposure using biomarkers, akin to lead tests in children [25,49]. The results can give a better understanding of children’s exposure to ETS, a potent asthma trigger, and provide a new opportunity for asthma education and intervention.
